# Heterologous Expression of the Barley (*Hordeum vulgare* L.) *Xantha-f, -g* and -*h* Genes that Encode Magnesium Chelatase Subunits

**DOI:** 10.1007/s10930-020-09913-0

**Published:** 2020-07-31

**Authors:** Rabab Mahdi, David Stuart, Mats Hansson, Helmy M. Youssef

**Affiliations:** 1 Department of Biology, Lund University, Sölvegatan 35, 22362 Lund, Sweden; 2 Faculty of Agriculture, Cairo University, Giza, 12613 Egypt

**Keywords:** Magnesium chelatase, Barley, Xantha, Chlorophyll biosynthesis, Mg-chelatase, Protoporphyrin

## Abstract

Biosynthesis of chlorophyll involves several enzymatic reactions of which many are shared with the heme biosynthesis pathway. Magnesium chelatase is the first specific enzyme in the chlorophyll pathway. It catalyzes the formation of Mg-protoporphyrin IX from the insertion of Mg^2+^ into protoporphyrin IX. The enzyme consists of three subunits encoded by three genes. The three genes are named *Xantha-h*, *Xantha-g* and *Xantha-f* in barley (*Hordeum vulgare* L.). The products of the genes have a molecular weight of 38, 78 and 148 kDa, respectively, as mature proteins in the chloroplast. Most studies on magnesium chelatase enzymes have been performed using recombinant proteins of *Rhodobacter capsulatus*, *Synechocystis* sp. PCC6803 and *Thermosynechococcus elongatus*, which are photosynthetic bacteria. In the present study we established a recombinant expression system for barley magnesium chelatase with the long-term goal to obtain structural information of this enigmatic enzyme complex from a higher plant. The genes *Xantha-h*, *-g* and -*f* were cloned in plasmid pET15b, which allowed the production of the three subunits as His-tagged proteins in *Escherichia coli* BL21(DE3)pLysS. The purified subunits stimulated magnesium chelatase activity of barley plastid extracts and produced activity in assays with only recombinant proteins. In preparation for future structural analyses of the barley magnesium chelatase, stability tests were performed on the subunits and activity assays were screened to find an optimal buffer system and pH.

## Introduction

One of the characteristic steps of chlorophyll biosynthesis is performed by the enzyme magnesium chelatase, which inserts Mg^2+^ into the tetrapyrrole protoporphyrin IX to produce Mg-protoporphyrin IX [1–4]. Early studies on barley (*Hordeum vulgare* L.) mutants suggested that the enzyme requires three gene products encoded by *Xantha-h*, *Xantha-g* and *Xantha-f* [5]. Later, characterization of a "photosynthetic gene cluster" of 45 kbp in the photosynthetic purple bacteria *Rhodobacter capsulatus* and *Rhodobacter sphaeroides* identified three genes named *bchI*, *bchD* and *bchH*, which are the structural genes of the three components of magnesium chelatase [6–9]. The magnesium chelatase genes in the cyanobacterium *Synechocystis* sp. PCC 6803 were also studied and named *chlI*, *chlD* and *chlH* [10, 11]. In order to avoid confusion, the genes are often referred to by their original names and the proteins as I, D and H corresponding to the gene products of *Xantha-h*/*bchI*/*chlI*, *Xantha-g*/*bchD*/*chlD* and *Xantha-f*/*bchH*/*chlH*, respectively. The size of the I, D and H subunits are typically in the range of 38–45 kDa, 60–85 kDa and 129–155 kDa, respectively. The specific size of the barley proteins are 38, 78 and 148 kDa as mature proteins after import into the chloroplast.

It was found early that the magnesium chelatase reaction is powered by hydrolysis of ATP [12, 13]. This is now understood in the view that the I and D subunits belong to the large group of AAA + proteins (ATPases Associated with various cellular Activities), which form a two-ringed structure with three dimers of I in one ring and three dimers of D in the other ring [14]. The H subunit has been suggested to be the catalytic subunit and the target of the ID complex [15]. During catalysis, ATP is specifically hydrolyzed by the I subunits. No ATP hydrolysis has been associated with the D subunit although the N-terminal half of the D subunit is very similar to the I subunit [11, 16].

Most biochemical and structural analyses of magnesium chelatase have been performed with proteins from *R. capsulatus*, *Thermosynechococcus elongatus* and *Synechocystis* sp. PCC 6803 (Table 1). In general, the three subunits are conserved when compared between different species. It can therefore be expected that the overall catalytic mechanism of magnesium chelatase is identical in all bacteriochlorophyll and chlorophyll containing organisms. Still, the I, D and H subunits of plants are distantly related to especially the corresponding proteins of *R. capsulatus*. To gain a better knowledge of plant magnesium chelatase we have in the present study established an expression system for the three barley magnesium chelatase genes in *Escherichia coli* and purified the recombinant subunits. The protein subunits showed magnesium chelatase activity and will be used in future studies of the magnesium chelatase structure.

**Table 1:** Published structural information concerning the AAA + class of chelatases

Organism	Subunit	Method	Tertiary structure	Reference
*R. capsulatus*	BchI	X-ray crystallography	Hexamer	[33]
*R. capsulatus*	BchI	Negative-stain EM	Hexamer	[34]
*R. capsulatus*	BchH	Negative-stain EM	Monomer	[35]
*R. capsulatus*	BchID complex	Cryo-EM	Trimer of dimers	[14, 36]
*B. melitensis*	CobST complex	Cryo-EM	Trimer of dimers	[37]
*T. elongates*	ChlH	Negative-stain EM, Small-angle X-ray scattering	Monomer	[38]
*T. elongates*	ChlH	Negative-stain EM	Monomer	[39]
*Synechocystis sp. PCC6803*	ChlI	Negative-stain EM	Heptamer	[40]
*Synechocystis sp. PCC6803*	ChlI	X-ray crystallography	Hexamer	[41]
*Synechocystis sp. PCC6803*	ChlH	X-ray crystallography	Dimer	[42]

*EM* electron microscopy

## Material and Methods

### General DNA Techniques

Clones of barley *Xantha-h, Xantha-g* and *Xantha-f* inserted into plasmid pET15b were ordered from GenScript (GenScript). Competent *E. coli* cells were prepared as described by Mandel and Higa [17]. Plasmids were maintained in *E. coli* XL1-Blue (Stratagene). Expression of genes cloned in pET15b were performed in *E. coli* BL21(DE3)pLysS. Plasmids were isolated with the Jet Quick® Plasmid DNA Purification Kit (Life Technologies Corporation).

### Expression of *Xantha-h, Xantha-g, Xantha-f*

*E. coli* BL21(DE3)pLysS was transform with plasmids and plated on Tryptose Blood Agar Base (Difco) containing chloramphenicol (25 μg/ml) and ampicillin (50 μg/ml), followed by overnight incubation at 37 °C. Transformants were collected and used to inoculate one liter of LB medium containing 25 mg/ml chloramphenicol and 100 mg/ml ampicillin to an optical density at 600 nm (OD_600_) of 0.05–0.1. The cultures were incubated at 30 °C on a rotary shaker (200 rpm). IPTG (isopropyl β-d-1-thiogalactopyranoside) was added to a concentration of 1 mM when the cultures had reached OD_600_ 0.5–0.7. Incubation continued overnight at 30 °C, except the culture with *E. coli* BL21(DE3)pLysS/pET15bXanF which was moved to 16 °C and incubated for 40 h. All handling of XanF during expression, purification and activity measurements were performed without direct input of light since the H-subunit of *R. capsulatus* is known to be light sensitive [18].

### Purification of Recombinant Proteins

Cells of *E. coli* producing recombinant XanH, XanG and XanF were harvested by centrifugation at 11,325×g for 20 min at 4 °C. Pellets were washed with binding buffer composed of 20 mM imidazole, 0.5 M NaCl, 20 mM Tris–HCl pH 8.0. Aliquots of cell pellets corresponding to 250 ml of cultures were stored at − 80 °C. Upon usage, a cell pellet was thawed and resuspended in binding buffer supplemented with 1 mM PMSF (phenylmethanesulfonyl fluoride) and 4 mM DTT (dithiothreitol). A French press at 16,000 psi (pounds per square inch) was used to lyse cells followed by centrifugation at 48,384×g, 20 min, 4 °C. XanH and XanF proteins were further purified from the soluble fractions. Similar to the *R. capsulatus* D-subunit, XanG formed inclusion bodies and was obtained in the pellet fraction. The XanG protein was solubilized from the pellet fraction according to the method to solubilize the *R. capsulatus* d-subunit [19]. The pellet containing XanG was resuspended in binding buffer containing 6 M urea and incubated for 1 h on ice before centrifugation at 5000×g for 20 min at 4 °C. XanG was further purified from the urea containing supernatant.

The recombinant XanH, XanG and XanF were purified by immobilized metal ion affinity chromatography (IMAC) using 1 ml HisTrap™ FF Crude columns (GE Healthcare). After loading the soluble fractions through the column, washing was performed with 10 column volumes of binding buffer followed by 10 column volumes of wash buffer composed of 40 mM imidazole, 0.5 M NaCl, 20 mM Tris–HCl pH 8.0. Protein was eluted with elution buffer composed of 250 mM imidazole, 0.5 M NaCl, 20 mM Tris–HCl pH 8.0. Six M urea was included in all buffers for purification of XanG. The eluted proteins were desalted with NAP-10 columns (GE Healthcare). XanH was desalted into 20 mM Tris–HCl pH 8.0. XanG was desalted into 6 M urea, 20 mM Tris–HCl pH 8.0. XanF was desalted into 50 mM Tricine-NaOH pH 8.0, 250 mM NaCl, 50 mM MgCl_2_. Protein concentrations were determined by Bradford Reagent (BioRad).

### Preparation of Barley Chloroplast Proteins

Seeds of barley cultivar Bonus were planted into two 50 × 60 cm trays in moist vermiculate and grown in darkness at room temperature. After eight days, etiolated yellow seedlings were illuminated for 4–5 h before harvest. The top 7 cm of the primary leaves were collected and homogenized in a blender with exchangeable razorblades in grinding medium composed of 0.4 M d-mannitol, 20 mM Tricine-NaOH pH 9.0 and 1 mM DTT. The mixture was then filtered through a double layer of nylon tissue and centrifuged at 2000×g, 4 °C, 5 min. The pellet was resuspended in 50 ml grinding medium and aliquots of 6 ml were layered on 8 ml Percoll cushions (40% Percoll [Sigma-Aldrich] and 1 mM DTT in grinding buffer) in 15 ml tubes and centrifuged at 3220×g, 4 °C, 40 min with a swing-out rotor. The pellet was resuspended in grinding medium, transferred to a fresh tube and centrifuged at 2000×*g*, 4 °C for 5 min. The plastids in the resulting pellet were resuspended and lysed in one ml of lysis buffer composed of 20 mM Tricine-NaOH pH 9.0, 1 mM DTT and 1 mM PMSF. Next, the lysate was ultracentrifuged at 100,000×*g*, 4 °C for 20 min using a fixed angle rotor. Supernatants were collected and glycerol was added to a final concentration of 10%. Aliquots were stored at − 80 °C and used in enzyme assays.

### Magnesium Chelatase Assays

Magnesium chelatase assays were performed in total volumes of 50 μl and contained 20 mM Tricine-NaOH pH 9.0, 4 mM ATP, 10 mM creatine phosphate, 15 mM MgCl_2_, 2 μM deuteroporphyrin IX, 2.5 units creatine phosphokinase and recombinant barley magnesium chelatase subunits. Assays were stopped after 30 min by addition of 950 μl acetone:water: 32% ammonia (80:20:1, vol:vol:vol) followed by centrifugation for 2 min at 17,000×g to remove precipitated protein. The assays were kept in dark during mixing and incubation to prevent photo-oxidation. The supernatants were analyzed on a Shimadzu RF-5301PC spectrofluorometer with excitation wavelength set at 408 nm and emission detected between 550 and 650 nm. Excitation and emission slit widths were 5 nm and 10 nm, respectively. The amount of formed Mg-deuteroporphyrin IX was determined from the peak at 585 nm. For testing the effect of different buffer systems 20 mM MOPS-NaOH (pH 6.5, 7.0 and 7.5), 20 mM Tris–HCl (pH 7.5, 8.0, 8.5 and 9.0), 20 mM Tricine-NaOH (pH 7.5, 8.0, 8.5 and 9.0), 20 mM HEPES–NaOH (pH 7.0, 7.5 and 8.0) and 20 mM CHES-NaOH (pH 8.5, 9.0, 9.5 and 10.0) were used.

### Phylogenetic Analysis

Barley XanH (GenBank: ABF72535.3), XanG (GenBank: AAZ32779.1) and XanF (GenBank: KAE8772866.1) polypeptide sequences were queried against UniProtKB (www.uniprot.org) to retrieve homologs from other species. Transit peptides were identified by ChloroP [20] and removed from the plant polypeptides before alignments were made. Protein sequences showing ≥ 40% identity to the query sequence were used for phylogenetic analysis. To perform phylogenetic analysis, protein sequences were initially aligned using the MUSCLE algorithm implemented in MEGAX with default setting [21]. A maximum likelihood phylogenetic tree was constructed using the maximum likelihood heuristic method named Nearest Neighbor Interchange implemented in MEGAX. The bootstrap consensus tree inferred from 1000 replicates is taken to represent the evolutionary history of the analyzed sequences. Branches which correspond to partitions replicated in bootstrap replicates of less than 50% collapse.

## Results

### Phylogenetic Analysis of Magnesium Chelatase Subunits

**Fig. 1:**
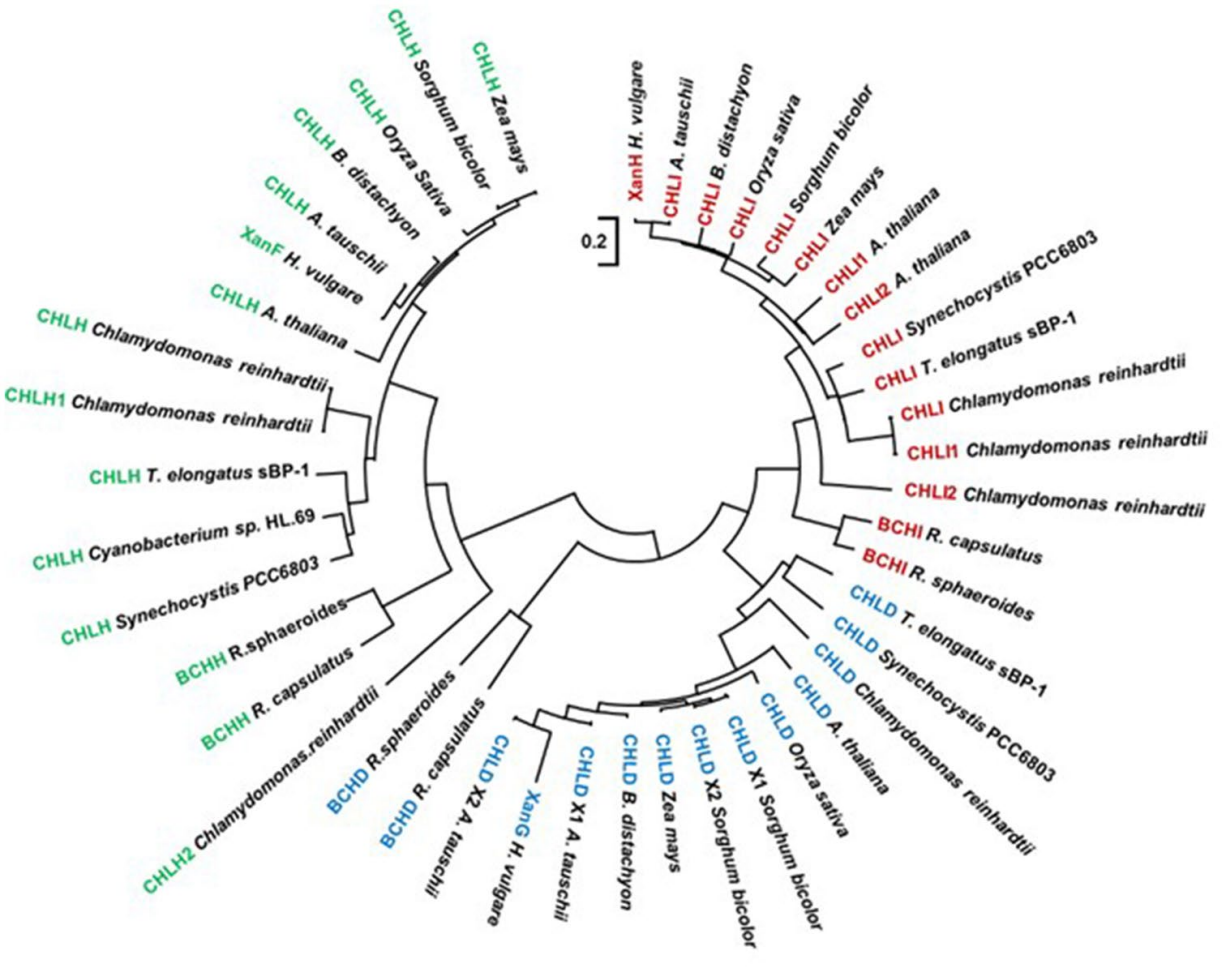
A maximum likelihood phylogenetic tree of magnesium chelatase subunits of different species; *Rhodobacter capsulatus*, *Rhodobacter sphaeroides*, *Thermosynechococcus elongatus*, *Synechocystis* PCC 6803, *Chlamydomonas reinhardtii*, *Cyanobacterium sp.* HL.69, *Arabidopsis thaliana*, *Oryza sativa*, *Sorghum bicolor*, *Zea mays*, *Brachypodium distachyon*, *Aegilops tauschii* and *Hordeum vulgare*. Three main separate clades are evident; one clade for each subunit

Magnesium chelatase occurs in a broad variety of photosynthetic species that use bacteriochlorophyll or chlorophyll as the light absorbing pigment. We selected 44 different proteins belonging to the three magnesium chelatase subunits and performed a phylogenetic analysis (Fig. 1). Within each clade corresponding to each subunit, the proteins of the photosynthetic purple bacterium *R. capsulatus* are the most divergent. The proteins of the cyanobacteria *T. elongatus* and *Synechocystis* are relatively closely related to each other. The magnesium chelatase proteins of higher plants are distinctly different to those of *R. capsulatus*, *T. elongatus* and *Synechocystis*. This encouraged us to set up a pipeline for production of barley magnesium chelatase in order to get insight of this protein complex in higher plants.

### Construction of Expression Plasmids

**Fig. 2:**
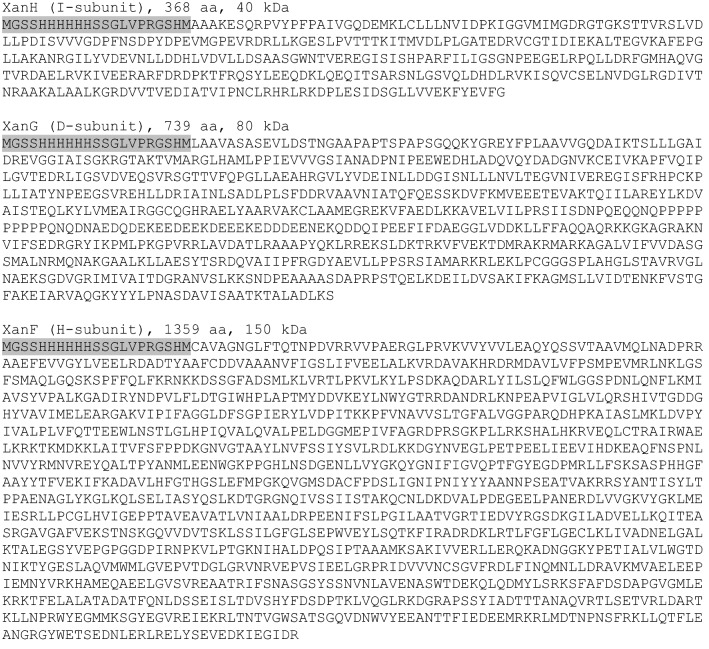
The polypeptide sequences of the barley magnesium chelatase subunits as produced from pET15bXanH, pET15bXanG and pET15bXanF. The sequence marked with grey is derived from pET15b and contains the His-tag

In order to obtain recombinant subunits of barley magnesium chelatase, *Xantha-h*, *-g* and *-f* were expressed from plasmid pET15b in *E. coli*, which resulted in N-terminally His-tagged proteins that could be purified with immobilized metal ion affinity chromatography (IMAC). We chose to use N-terminal His-tags, which have been successful in most magnesium chelatase expression systems. C-terminal His-tags have sometimes been used for the I subunit since an early report found that an N-terminal His-tag on the I subunit was inhibitory [19]. In barley and other plants, the magnesium chelatase subunits are imported into the chloroplast with help of a transit peptide. ChloroP [20] was used to predict the transit peptide sequence of the three subunits. The genes, corresponding to the mature proteins, were ordered from GenScript and cloned between the *Nde*I–*Xho*I restriction sites of pET15b. The synthetic genes were codon optimized for expression in *E. coli*. The expression plasmids were named pET15bXanH, pET15bXanG and pET15bXanF and the resulting polypeptides are shown in Fig. 2.

### Production and Purification of Barley Magnesium Chelatase Subunits

**Fig. 3:**
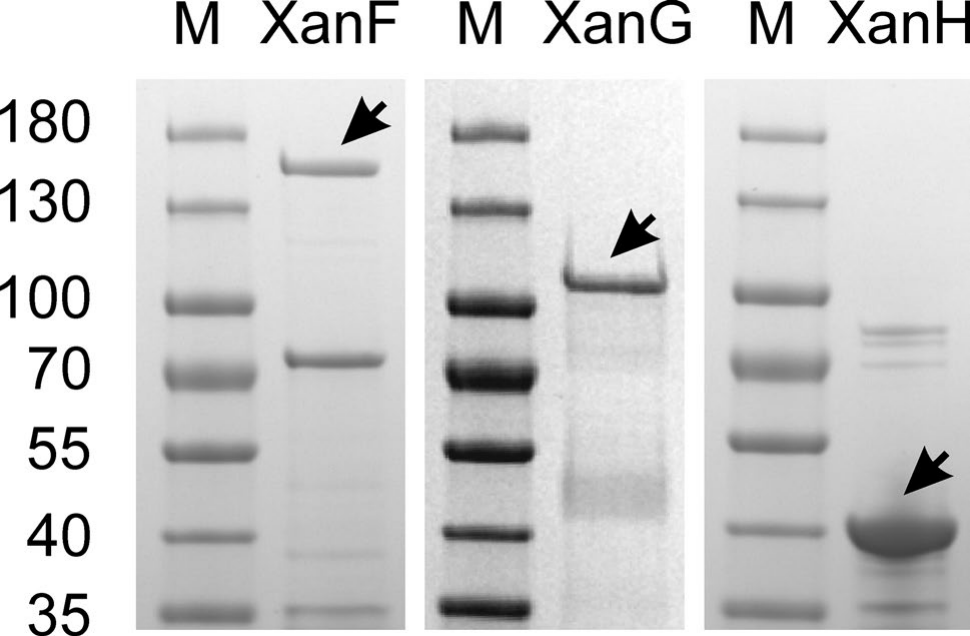
SDS–polyacrylamide gel of XanF, XanG and XanH after purification and desalting. The expected molecular mass of the respective protein is 150, 80 and 40 kDa including the N-terminal His-tag. XanG migrates like a 105 kDa protein. M; Migration positions of standard proteins with the indicated molecular masses (in kDa)

The barley magnesium chelatase genes were expressed in *E. coli* BL21(DE3)pLysS from plasmids pET15bXanH, pET15bXanG and pET15bXanF. *E. coli* BL21(DE3) was less efficient (data not shown). XanG formed inclusion bodies, which had to be solubilized with urea. This is also the case for the *R. capsulatus* ortholog [19]. Thus, all buffers used for purification of XanG contained 6 M urea. Also, XanH and XanF tended to form inclusion bodies but enough protein remained in supernatant fractions after centrifugation for further purification. The subunits were purified by IMAC followed by desalting. Typically, 1–8 mg of protein were obtained from 250 ml of bacterial culture (Fig. 3).

### Active Recombinant Barley Magnesium Chelatase Subunits

**Fig. 4:**
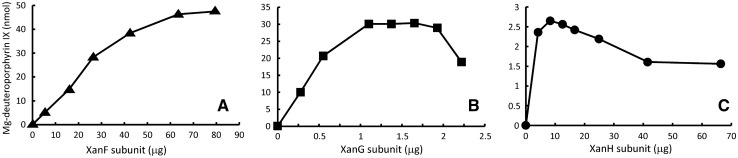
Measurements of magnesium chelatase activity with recombinant barley protein subunits XanH, XanG and XanF. In the three experiments two subunits were kept at constant amounts and the amount of the third subunit was varied. **a** 42 µg (1.0 nmol) XanH, 1.1 µg (14 pmol) XanG and 0–80 µg XanF. **b** 42 µg XanH, 0–2.5 µg XanG and 27 µg (180 pmol) XanF. **c** 0–70 µg XanH, 1.1 µg XanG and 27 µg XanF

Magnesium chelatase activity can be measured with protein extract isolated from barley chloroplasts [22]. In the current study we prepared such plastid extract, which showed magnesium chelatase activity when used in enzymatic assays demonstrating that all magnesium chelatase subunits are present in the plastid extract. We hypothesized that addition of any of the three barley magnesium chelatase subunits produced in *E. coli* as recombinant proteins would further increase the enzymatic activity. Thus, combining the barley plastid extract with fractions from the heterologous expression system in *E. coli* allowed us to work with one expression system at the time until an active recombinant magnesium chelatase subunit was obtained. Therefore, various purification fractions of recombinant XanF, XanG or XanH were added to magnesium chelatase assay mixtures containing barley plastid extract and a stimulation of activity indicated that an active recombinant subunit had been prepared (data not shown). When all three subunits showed activity in these assays, they were successfully combined with each other without plastid extracts. In different assays, the amount of two subunits were kept constant and the third unit was varied (Fig. 4). In our current model of the enzymatic mechanism based on the *R. capsulatus* magnesium chelatase, the D subunit forms a hexameric platform for the stepwise assembly of the I subunits into a two-tiered hexameric ring [3]. The model explains the observation that an excess of the D subunit over the I subunit lowers the activity [16]. In contrast, increasing amounts of I and H subunits lead to saturation of these subunits and hyperbolic curves. Our initial experiments with the barley magnesium chelatase subunits suggest that they follow the same enzymatic mechanism (Fig. 4).

### Finding Optimal Conditions for Barley Magnesium Chelatase

**Fig. 5:**
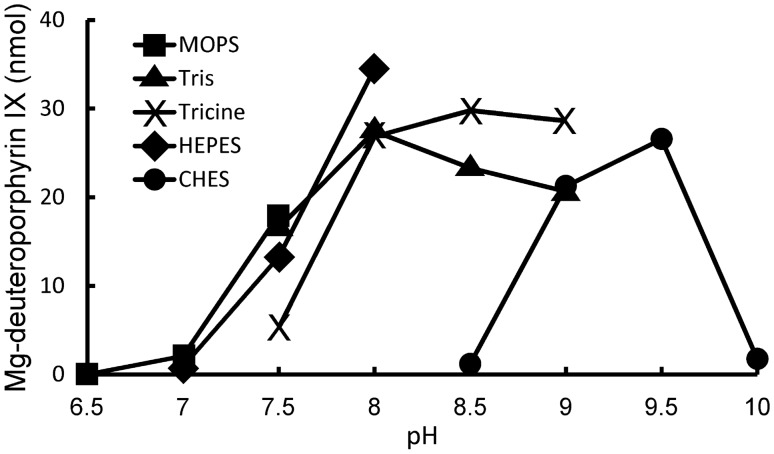
Barley magnesium chelatase assays performed with different buffer systems and pH. Amounts of used proteins in each assay: XanH (16.6 µg), XanG (1.1 µg) and XanF (26 µg)

Since structural analysis is the long-term goal of our work with the barley magnesium chelatase, it was important to obtain basic information about optimal buffer systems and storage conditions. MOPS, Tris, Tricine, HEPES and CHES where used to cover different pH ranges and used in magnesium chelatase assays with the recombinant subunits. The barley magnesium chelatase appeared to be relatively tolerant regarding the range of pH. Good activity was obtained in all buffer systems between pH 8.0–9.5, with the exception of CHES at pH 8.5 (Fig. 5).

**Fig. 6:**
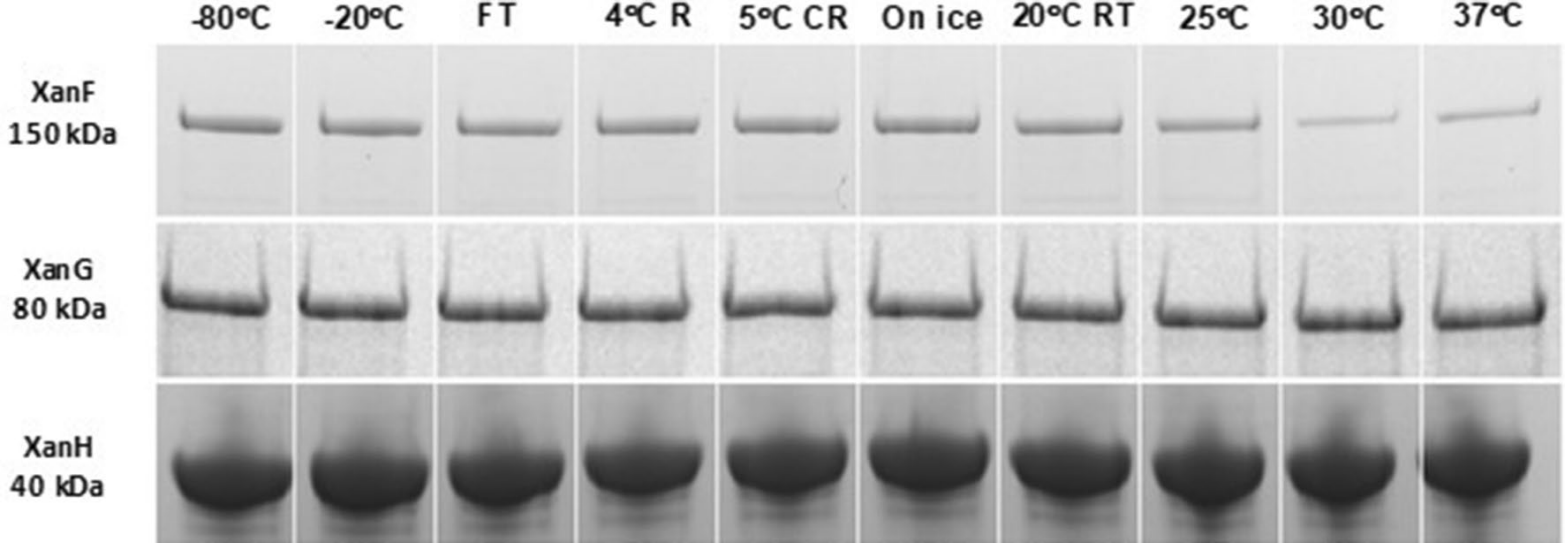
Thermal stability test of recombinant barley magnesium chelatase subunits. The proteins were kept at different temperatures for 24 h and then analyzed by SDS-PAGE. 83 µg XanH, 11 µg XanG and 53 µg XanF were used in each experiment. Lane 1, -80 °C; 2, -20 °C; 3, -20 °C and freeze thawed one time; 4, 4 °C (refrigerator); 5. 4 °C (cold room); 6, 0° (ice); 7, 20 °C; 8, 25 °C; 9, 30 °C; 10, 37 °C

The purified subunits were tested for their thermal stability by exposing them to different storage conditions over 24 h. The temperatures varied from − 80 to 37 °C and also included repeated freeze thawing of a samples stored at − 20 °C. After 24 h the samples were analyzed by SDS-PAGE. In general, the subunits withstood the treatments. The only negative effect was seen in the case of XanF stored at 30 and 37 °C (Fig. 6).

## Discussion

Chlorophyll and heme are the most abundant tetrapyrroles in biological systems. They share the first initial catalytic steps of the tetrapyrrole biosynthetic pathway [4, 23]. A major difference between them is the bound metal ion, which is magnesium in chlorophyll and iron in heme. Protoporphyrin IX is their last common intermediate and used by magnesium chelatase or ferrochelatase for the insertion of Mg^2+^ or Fe^2+^, respectively. Although the porphyrin substrate is the same, the two enzymes show no obvious similarities. While ferrochelatase is a monomer or homodimer, magnesium chelatase is a large protein complex composed of three gene products and requires ATP for catalysis [24]. Interestingly, there are two different cobalt chelatases involved in synthesis of cobalamin (vitamin B_12_). The cobalt chelatase participating in the anaerobic pathway is structurally similar to ferrochelatase, while the cobalt chelatase involved in the aerobic pathway belongs to the AAA + protein family like magnesium chelatase and requires input of energy in the form of ATP [25]. Today, the structural information available for the AAA + class of chelatases is obtained from a limited group of organisms; *R. capsulatus*, *Synechocystis* sp. PCC 6803, *T. elongatus* and *Brucella melitensis* (Table 1). We would like to obtain structural insight into the magnesium chelatase of plants and therefore established an expression system of the barley magnesium chelatase genes since barley has a long history in the field of magnesium chelatase research. The oldest mutant in barley, *xantha-f.10*, was induced 1953 by X-ray in the cultivar Bonus [26]. A total of 20 barley mutants are available and the mutations have been described at DNA level [27–31]. The barley magnesium chelatase proteins are most different from those of *R. capsulatus*, which is obvious from a phylogenetic analysis of the three subunits (Fig. 1). *R. capsulatus* produces bacteriochlorophyll under anaerobic conditions and the differences in the polypeptide sequences are probably adaptations to an anaerobic environment [32]. In alignments between the *R. capsulatus* D polypeptide to the D subunits of plants, several gaps are found [3]. Several gaps are also found in alignments of H subunits but the *R. capsulatus* H sequence has also an addition of an iron-sulfur cluster sequence that has been suggested to be involved in regulation of magnesium chelatase activity in this bacterium [32]. We hope that the present heterologous expression system of barley magnesium chelatase will allow us to perform studies on a plant magnesium chelatase and reveal plant specific information of this key enzyme of chlorophyll biosynthesis.

## References

[1] Willows RD, Hansson M, Kadish KM, Smith KM, Guilard R (2003). Mechanism, structure, and regulation of magnesium chelatase. The porphyrin handbook: chlorophylls and bilins: biosynthesis, synthesis and degradation.

[2] Kannangara CG, von Wettstein D, Rebeiz CA (2010). Magnesium chelatase. The chloroplast. Advances in photosynthesis and respiration.

[3] Hansson M, Lundqvist J, Sirijovski N, Al-Karadaghi S, Ferreira GC, Kadish KM, Smith KM, Guilard R (2014). Magnesium chelatase: The molecular motor of chlorophyll biosynthesis. Handbook of porphyrin science with applications to chemistry, physics, materials science, engineering, biology and medicine (Vol 28, pp 41–84).

[4] Willows RD (2019). The Mg branch of chlorophyll synthesis: biosynthesis of chlorophyll *a* from protoporphyrin IX. Adv Bot Res.

[5] von Wettstein D, Kahn A, Nielsen OF, Gough S (1974). Genetic regulation of chlorophyll synthesis analyzed with mutants in barley. Science.

[6] Marrs B (1981). Mobilization of the genes for photosynthesis from *Rhodopseudomonas capsulata* by a promiscuous plasmid. J Bacteriol.

[7] Bollivar DW, Suzuki JY, Beatty JT (1994). Directed mutational analysis of bacteriochlorophyll *a* biosynthesis in *Rhodobacter capsulatus*. J Mol Biol.

[8] Gibson LC, Willows RD, Kannangara CG, von Wettstein D, Hunter CN (1995). Magnesium-protoporphyrin chelatase of *Rhodobacter sphaeroides*: reconstitution of activity by combining the products of the *bchH*, *-I*, and *-D* genes expressed in *Escherichia coli*. Proc Natl Acad Sci USA.

[9] Willows RD, Gibson LCD, Kanangara CG, Hunter CN, von Wettstein D (1996). Three separate proteins constitute the magnesium chelatase of *Rhodobacter sphaeroides*. Eur J Biochem.

[10] Jensen PE, Gibson LCD, Henningsen KW, Hunter CN (1996). Expression of the *chlI, chlD*, and *chlH* genes from the cyanobacterium *Synechocystis* PCC6803 in *Escherichia coli* and demonstration that the three cognate proteins are required for magnesium-protoporphyrin chelatase activity. J Biol Chem.

[11] Farmer DA, Brindley AA, Hitchcock A, Jackson PJ, Johnson B, Dickman MJ, Hunter CN, Reid JD, Adams NBP (2019). The ChlD subunit links the motor and porphyrin binding subunits of magnesium chelatase. Biochem J.

[12] Gorchein A (1973). Control of magnesium–protoporphyrin chelatase activity in *Rhodopseudomonas spheroides*. Role of light, oxygen, and electron and energy transfer. Biochem J.

[13] Castelfranco PA, Weinstein JD, Schwarcz S, Pardo AD, Wezelman BE (1979). The Mg insertion step in chlorophyll biosynthesis. Arch Biochem Biophys.

[14] Lundqvist J, Elmlund H, Peterson Wulff R, Berglund L, Elmlund D, Emanuelsson C, Hebert H, Willows RD, Hansson M, Lindahl M, Al-Karadaghi S (2010). ATP-induced conformational dynamics in the AAA+ motor unit of magnesium chelatase. Structure.

[15] Sawicki A, Willows RD (2008). Kinetic analyses of the magnesium chelatase provide insights into the mechanism, structure, and formation of the complex. J Biol Chem.

[16] Axelsson E, Lundqvist J, Sawicki A, Nilsson S, Schröder I, Al-Karadaghi S, Willows RD, Hansson M (2006). Recessiveness and dominance in barley mutants deficient in Mg-chelatase subunit D, an AAA protein involved in chlorophyll biosynthesis. Plant Cell.

[17] Mandel M, Higa A (1970) Calcium-dependent bacteriophage DNA infection. J Mol Biol 53:159–162 10.1016/0022-2836(70)90051-3 4922220

[18] Sirijovski N, Olsson U, Lundqvist J, Al-Karadaghi S, Willows RD, Hansson M (2006). ATPase activity associated with the magnesium chelatase H-subunit of the chlorophyll biosynthetic pathway is an artefact. Biochem J.

[19] Willows RD, Beale SI (1998). Heterologous expression of the *Rhodobacter capsulatus BchI*, *-D*, and *-H* genes that encode magnesium chelatase subunits and characterization of the reconstituted enzyme. J Biol Chem.

[20] Emanuelsson O, Nielsen H, von Heijne G (1999). ChloroP, a neural network-based method for predicting chloroplast transit peptides and their cleavage sites. Protein Sci.

[21] Kumar S, Stecher G, Li M, Knyaz C, Tamura K (2018). MEGA X: molecular evolutionary genetics analysis across computing platforms. Mol Biol Evol.

[22] Kannangara CG, Vothknecht UC, Hansson M, von Wettstein D (1997). Magnesium chelatase: association with ribosomes and mutant complementation studies identify barley subunit Xantha-G as a functional counterpart of *Rhodobacter* subunit BchD. Mol Gen Genet.

[23] Tanaka R, Tanaka A (2007). Tetrapyrrole biosynthesis in higher plants. Annu Rev Plant Biol.

[24] Adams NB, Brindley AA, Hunter CN, Reid JD (2016). The catalytic power of magnesium chelatase: a benchmark for the AAA(+) ATPases. FEBS Lett.

[25] Schubert HL, Raux E, Wilson KS, Warren MJ (1999). Common chelatase design in the branched tetrapyrrole pathways of heme and anaerobic cobalamin synthesis. Biochem.

[26] Henningsen KW, Boynton JE, von Wettstein D (1993). Mutants at *xantha* and *albina* loci in relation to chloroplast biogenesis in barley (*Hordeum vulgare* L.). The Royal Danish Academy of Sciences and Letters.

[27] Jensen PE, Petersen BL, Stummann BM, Henningsen KW, Willows RD, Vothknecht UC, Kannangara CG, von Wettstein D (1996). Structural genes for Mg-chelatase subunits in barley: *Xantha-f, -g and -h*. Mol Gen Genet.

[28] Hansson A, Kannangara CG, von Wettstein D, Hansson M (1999). Molecular basis for semidominance of missense mutations in the XANTHA-H (42-kDa) subunit of magnesium chelatase. Proc Natl Acad Sci USA.

[29] Hansson A, Willows RD, Roberts TH, Hansson M (2002). Three semidominant barley mutants with single amino acid substitutions in the smallest magnesium chelatase subunit form defective AAA+ hexamers. Proc Natl Acad Sci USA.

[30] Braumann I, Stein N, Hansson M (2014). Reduced chlorophyll biosynthesis in heterozygous barley magnesium chelatase mutants. Plant Physiol Biochem.

[31] Olsson U, Sirijovski N, Hansson M (2004). Characterization of eight barley *xantha-f* mutants deficient in magnesium chelatase. Plant Physiol Biochem.

[32] Sirijovski N, Mamedov F, Olsson U, Styring S, Hansson M (2007). *Rhodobacter capsulatus* magnesium chelatase subunit BchH contains an oxygen sensitive iron-sulfur cluster. Arch Microbiol.

[33] Fodje MN, Hansson A, Hansson M, Olsen JG, Gough S, Willows RD, Al-Karadaghi S (2001). Interplay between an AAA module and an integrin I domain may regulate the function of magnesium chelatase. J Mol Biol.

[34] Willows RD, Hansson A, Birch D, Al-Karadaghi S, Hansson M (2004). EM single particle analysis of the ATP-dependent BchI complex of magnesium chelatase: an AAA(+) hexamer. J Struct Biol.

[35] Sirijovski N, Lundqvist J, Rosenbäck M, Elmlund H, Al-Karadaghi S, Willows RD, Hansson M (2008). Substrate-binding model of the chlorophyll biosynthetic magnesium chelatase BchH subunit. J Biol Chem.

[36] Elmlund H, Lundqvist J, Al-Karadaghi S, Hansson M, Hebert H, Lindahl M (2008). A new cryo-EM single-particle *ab initio* reconstruction method visualizes secondary structure elements in an ATP-fueled AAA+ motor. J Mol Biol.

[37] Lundqvist J, Elmlund D, Heldt D, Deery E, Söderberg C, Hansson M, Warren M, Al-Karadaghi S (2009). The AAA(+) motor complex of subunits CobS and CobT of cobaltochelatase visualized by single particle electron microscopy. J Struct Biol.

[38] Qian P, Marklew CJ, Viney J, Davison PA, Brindley AA, Söderberg C, Al-Karadaghi S, Bullough PA, Grossmann JG, Hunter CN (2012). Structure of the cyanobacterial magnesium chelatase H subunit determined by single particle reconstruction and small-angle x-ray scattering. J Biol Chem.

[39] Adams NBP, Marklew CJ, Qian P, Brindley AA, Davison PA, Bullough PA, Hunter CN (2014). Structural and functional consequences of removing the N-terminal domain from the magnesium chelatase ChlH subunit of *Thermosynechococcus elongatus*. Biochem J.

[40] Reid JD, Siebert CA, Bullough PA, Hunter CN (2003). The ATPase activity of the ChlI subunit of magnesium chelatase and formation of a heptameric AAA_ ring. Biochemistry.

[41] Gao Y-S, Wang Y-L, Wang X, Liu L (2019). Hexameric structure of the ATPase motor subunit of magnesium chelatase in chlorophyll biosynthesis. Protein Sci.

[42] Chen X, Pu H, Fang Y, Wang X, Zhao S, Lin Y, Zhang M, Dai H-E, Gong W, Liu L (2015). Crystal structure of the catalytic subunit of magnesium chelatase. Nature Plants.

